# Effect of genotyping errors on linkage map construction based on repeated chip analysis of two recombinant inbred line populations in wheat (*Triticum aestivum* L.)

**DOI:** 10.1186/s12870-024-05005-8

**Published:** 2024-04-22

**Authors:** Xinru Wang, Jiankang Wang, Xianchun Xia, Xiaowan Xu, Lingli Li, Shuanghe Cao, Yuanfeng Hao, Luyan Zhang

**Affiliations:** State Key Laboratory of Crop Gene Resources and Breeding, Institute of Crop Sciences, Chinese Academy of Agricultural Sciences (CAAS), Beijing, 100081 China

**Keywords:** Genotyping error, Linkage map, Error correction, Simulation study

## Abstract

Linkage maps are essential for genetic mapping of phenotypic traits, gene map-based cloning, and marker-assisted selection in breeding applications. Construction of a high-quality saturated map requires high-quality genotypic data on a large number of molecular markers. Errors in genotyping cannot be completely avoided, no matter what platform is used. When genotyping error reaches a threshold level, it will seriously affect the accuracy of the constructed map and the reliability of consequent genetic studies. In this study, repeated genotyping of two recombinant inbred line (RIL) populations derived from crosses Yangxiaomai × Zhongyou 9507 and Jingshuang 16 × Bainong 64 was used to investigate the effect of genotyping errors on linkage map construction. Inconsistent data points between the two replications were regarded as genotyping errors, which were classified into three types. Genotyping errors were treated as missing values, and therefore the non-erroneous data set was generated. Firstly, linkage maps were constructed using the two replicates as well as the non-erroneous data set. Secondly, error correction methods implemented in software packages QTL IciMapping (EC) and Genotype-Corrector (GC) were applied to the two replicates. Linkage maps were therefore constructed based on the corrected genotypes and then compared with those from the non-erroneous data set. Simulation study was performed by considering different levels of genotyping errors to investigate the impact of errors and the accuracy of error correction methods. Results indicated that map length and marker order differed among the two replicates and the non-erroneous data sets in both RIL populations. For both actual and simulated populations, map length was expanded as the increase in error rate, and the correlation coefficient between linkage and physical maps became lower. Map quality can be improved by repeated genotyping and error correction algorithm. When it is impossible to genotype the whole mapping population repeatedly, 30% would be recommended in repeated genotyping. The EC method had a much lower false positive rate than did the GC method under different error rates. This study systematically expounded the impact of genotyping errors on linkage analysis, providing potential guidelines for improving the accuracy of linkage maps in the presence of genotyping errors.

**Supplementary Information:**

The online version contains supplementary material available at 10.1186/s12870-024-05005-8.

## Introduction

Genotyping classifies life individuals to determine the linkage combination of genes, DNA sequences or genetic markers on chromosomes, according to allelic variations. Advances in sequencing-based genotyping technologies have allowed the genotyping for a large number of single nucleotide polymorphisms (SNP) loci in multiple individuals [1]. With marker number increased greatly, marker density augments accordingly. At the same time, map length is also exaggerated. One important reason for the length expansion is the presence of genotyping errors.

More and more researchers have realized that molecular analysis and manual sampling process are not fully reliable, and each step of genotyping process as well as various factors may produce genotyping errors [2, 3]. The major cause of genotyping error is effects of DNA sequence, low quantity or poor quality DNA, biochemical equipment and products, and human factors [4]. Genotyping errors may vary from experiment to experiment, so it is often overlooked in many scientific studies. However, even a moderate number of genotyping errors may dominate the accuracy of linkage studies [5–9]. For example, genotyping error rate of 1% can result in the loss of 21–58% of the linkage information for the situations simulated by [5].

Genotyping error may mask the true segregation of alleles, which has a serious impact on genetic studies, such as genetic linkage map construction, gene mapping, genomic selection and prediction. Construction of high-density and accurate linkage maps is an important field of genetic research. As early as the 1990s, it was shown that genotyping error can lead to incorrect map order and map length inflation. Each 1% error in a marker added 2 cM of inflation distance to the map, if there was one marker every 2 cM on average. In other words, an average error rate of 1% would double the map length [10, 11]. Effect of genotyping errors on linkage map construction can be explained by the decrease in accuracy of recombination frequency estimation. When a marker is located at both ends of one chromosome, each genotyping error causes one cross event. When a marker is located in the middle of one chromosome, each genotyping error causes two cross events. The more missing markers or genotyping errors a population has, the lower the accuracy of sequencing is observed [12, 13]. Quantitative trait locus (QTL) mapping is the process to determine the location of genetic loci for quantitative traits on chromosomes and estimate their genetic effects. Linkage disequilibrium (LD) between a QTL and a marker or a linear combination of markers is an important factor affecting the accuracy of QTL mapping [14]. Even a low genotyping error rate can have a far-reaching impact on LD measurement. With the increase of genotyping errors, the accuracy of LD estimation will decrease substantially. Effect of genotyping errors on genomic prediction is different under diverse genetic structures. Definitely, genomic prediction accuracy decreases with the increase of genotyping error rate, and the highest accuracy of genomic prediction is observed at error rate of zero and high heritability [15].

In recently years, researchers have conducted a series of studies to minimize the impact of genotyping errors. For example, genotyping error can be evaluated by genotyping repetitive samples and testing whether they deviate from Hardy-Weinberg equilibrium [16, 17]. It can also be determined by checking whether the marker data conforms to the Mendelian inheritance, the double recombination events of closely linked markers, and the consistency of repeated genotypes [18]. In fact, the real error rate is higher than the estimated value, which may be due to the “Mendelian compatibility” error, i.e., the wrong genotype may still conform to the Mendel’s laws of inheritances. Because of the various error types and different effects of each error type on the results, many algorithms and software packages for genotyping error detection and correction have been developed. For example, Genocheck [19], Pedcheck [20], MENDEL [21], SIMWALK [22], R/QTL [23], SOLOMON [24], GIGI-Check [25] can be used to detect Mendelian errors. LINKPHASE3 relies on the Mendelian segregation law to reconstruct haplotypes and correct genotyping errors [26]. ConGenR rapidly determines consensus genotypes and estimates genotyping errors from replicated genetic samples [27]. Smooth and Smooth-Descent predict genotyping errors, which improve the map quality and correctness of marker sequence [28, 29].

Main consequences of genotyping errors on map construction are the incorrect map order and map length expansion. In this study, repeated genotyping of two recombinant inbred line (RIL) populations derived from crosses Yangxiaomai × Zhongyou 9507 (YZ) and Jingshuang 16 × Bainong 64 (JB) using 15 K wheat Affymetrix SNP array in wheat were taken as examples to investigate the effect of genotyping errors on linkage map construction. Accuracy of different software packages for error correction was compared by using the two populations and simulated genotypic data with different levels of random errors. These findings not only specify an effective evaluation system of genotyping quality, but also provide an efficient approach to reduce the adverse effect of genotyping errors on the accuracy and reliability of linkage map construction.

## Materials and methods

### Plant materials and genotypic data

The two wheat populations used in this study were YZ F_6_ RILs and JB F_6_ RILs, which had been reported in Li et al. [30] and Xu et al. [31], respectively. The parents and 193 progenies in the YZ population (denoted as YZ1 to YZ193) were planted at Beijing and Shijiazhuang (Hebei Province) in 2011–2012 cropping season, and Gaoyi (Hebei Province) and Xinxiang (Henan Province) in 2019–2020 cropping season [30]. The parents and 181 progenies in the JB population (denoted as JB1 to JB181) were planted at Beijing and Gaoyi (Hebei Province) in 2019–2020 cropping season [31]. The samples for genotyping were harvested in Gaoyi 2019–2020 cropping season for both populations. Each population was genotyped twice at the same time by the 15 K wheat Affymetrix SNP array at China GoldenMarker (Beijing) Biotech Co., Ltd. (http://www.cgmb.com.cn/). Quality control was conducted on the genotypic data, by removing heterozygous and non-polymorphic markers in parents, and non-polymorphic markers in progenies. Common markers of the two replications of genotyping after quality control were filtrated and regarded as the original data (Supplemental Data 1 and 2 for the YZ population, and Supplemental Data 3 and 4 for the JB population). The YZ and JB populations had 4273 and 4497 SNP markers, respectively. These two data sets were denoted as data set 1 for YZ and data set 2 for JB, each with two replications (Table 1).

**Table 1:** Description for data sets used in this study

Data set	Description	Pop.^a^	Rep.^b^
1	Original genotypes after quality control	YZ	2
2	Original genotypes after quality control	JB	2
3	Genotypes corrected by the non-erroneous method	YZ	1
4	Genotypes corrected by the non-erroneous method	JB	1
5–14	5-50% individuals with a step size of 5% were randomly selected for repeated sequencing, each with 3 replications	JB	2
15	Genotypes corrected by the EC method	YZ	2
16	Genotypes corrected by the EC method	JB	2
17	Genotypes corrected by the GC method	YZ	2
18	Genotypes corrected by the GC method	JB	2

^a^ Population name, where YZ stands for Yangxiaomai × Zhongyou 9507 RIL population, and JB stands for Jingshuang 16 × Bainong 64 population

^b^ Number of replications of genotyping

### Calculation of missing and error rates

Consistent genotypes in the two replications of genotyping were treated as correct genotypes, while inconsistent genotypes were treated as genotyping errors. Missing and error rates of genotypes in the two RIL populations were calculated using R software by the following procedure. Firstly, missing marker points in one replication were also set as missing in the other replication to make missing points consistent between the two replications. Secondly, genotyping errors were classified into three types, i.e., 01, 02 and 12 errors, where the numbers 2, 1 and 0 represent the first parental, hybrid and the second parental genotypes, respectively. Error 01 meant that the genotype was 0 in one replication and 1 in the other replication. Similarly define 02 and 12 errors. Missing rate, error rate of each type, and total error rate were calculated in each population. Then, genotyping errors were replaced by missing values to obtain the non-erroneous genotypes. In other words, two replications of genotyping resulted in one set of non-erroneous genotypes, by replacing all inconsistent genotypes with missing values. The inconsistent genotypes included 01, 02, 12 errors and missing genotypes in one replication of genotyping. This treatment was named by the non-erroneous method for simplification. The resulted data sets were denoted as data set 3 for YZ and data set 4 for JB, each with one set of genotypic data (Table 1).

### Sampling of repeated genotyping individuals

In the present study, all RILs were genotyped twice and had repeated genotypes. Non-erroneous genotypes were obtained by applying the non-erroneous method on the two replications of genotyping. When the proportion of repeated genotyping individuals was lower, the genotypes achieved by the non-erroneous method still contained some errors. To study the impact of repeated proportion on linkage analysis, the JB population was taken as an example. A plug-in in EXCEL called square grid was used to randomly select 5-50% individuals with a step size of 5%, and each level was repeated for three times. The principle of not-putting-back random sampling was adopted. The sampled individuals were regarded as repeated genotyped, and then the non-erroneous method was applied. Genotypes of the other individuals had no treatment. In other words, 10 groups of genotypic data were generated by randomly sampling 5-50% repeated genotyping individuals. Each group contained three replications of sampling, and each sampling contained two replications of genotyping. The resulted data sets were denoted as data sets 5 to 14, corresponding to the 10 levels of repeated proportion (Table 1).

### Error detection by software packages

Besides the non-erroneous method, the accuracy of error detection by the two software packages were compared in the two populations. The first package is QTL IciMapping V4.2 [32]. We implemented an algorithm for error correction in QTL IciMapping, denoted as EC for short. For each marker point, theoretical frequency (*p*) of its genotype is calculated based on the genotypes of its neighboring markers and recombination frequencies between the three markers, which is also related to the population type and marker categories. Then a random number (*rn*) is generated between 0 and 1. If *rn* is larger than *p*, this marker point is regarded as a genotyping error, and then is replaced by missing values. Apply the EC method for the two replications of genotyping, respectively. The resulted data sets were denoted as data set 15 for YZ and data set 16 for JB, each with two replications (Table 1). The other package is Genotype-Corrector implemented by Python language and denoted as GC for short [33]. Specify the cutoff_SNP option to delete tags with missing rate higher than 80%. Use the sig_cutoff option to remove markers with severe singular separation. Merge the same homozygous markers in short genome interval of heterozygous region, set the sliding window size at 15, and then enter the process of genotype inference. Apply the GC method for the two replications of genotyping, respectively. The resulted data sets were denoted as data set 17 for YZ and data set 18 for JB, each with two replications (Table 1).

### Genetic linkage map construction

The MAP functionality in QTL IciMapping was used for linkage map construction on the 18 data sets described above. Method nnTwoOpt proposed by Zhang et al. [13] was adopted for marker ordering, which was a modifications of the k-Optimal (K-Opt) algorithm for solving the traveling-salesman problem (TSP). The other parameters were set as default. Pearson correlation coefficient between the linkage and physical maps was calculated for each constructed map by R software. Data sets 1 to 4 were also ordered by physical map to reflect the impact of genotyping error on recombination frequency estimation.

### Simulation study

To further explore the influence of genotyping errors on linkage analysis and efficiency of error correction methods, simulation experiments were designed with different levels of error rate. The BIP functionality in QTL IciMapping was used to simulate the genotypic data of one chromosome with markers evenly distributed. The marker density was set at 1 cM. Two marker numbers were considered, i.e. 100 and 200, corresponding to chromosome length of 100 and 200 cM. Five levels of genotyping error were randomly added into the simulated genotypes, i.e., 0.5%, 1%, 2%, 3%, and 5%. The EC and GC methods were adopted for genotyping error detection, respectively. Then the MAP functionality was used for linkage map construction on the simulated chromosome with errors as well as the corrected genotypic data. Each scenario in the simulation was repeated for 10 times, and the resulted map length was averaged from the 10 runs.

## Results

### Missing and error rates in the two RIL populations

The YZ population had missing rate of 1.18% and error rate of 0.35%, lower than the JB population (Tables S1, S2). In the YZ population, rates of 01, 02 and 12 errors were 0.23, 0.00 and 0.12%, respectively. Error rate was the highest on chromosome 7D, and lowest on chromosome 2D, whereas missing rate was the highest on chromosome 3D, and lowest on chromosome 6B (Table S1). Genotypic data of the JB population had missing rate of 1.42% and error rate of 8.47% (Table S2). Rates of 01, 02 and 12 errors were 3.09, 2.31 and 3.07%, respectively. Missing rate was the highest on chromosome 6D, and lowest on chromosome 4 A. Error rate was the highest on chromosome 3 A, and lowest on chromosome 1D.

### Comparison of genetic maps constructed using original genotypic data and non-erroneous genotype

The distribution of SNPs and linkage map information using the original data and non-erroneous genotypes were given in Table 2 for the YZ population and in Table 3 for the JB population. For population YZ, the full genome ordered by nnTwoOpt was 3940.48, 3930.33 and 3892.17 cM in length for replicate 1, replicate 2 and non-erroneous genotypes (Table 2). Chromosome length from the non-erroneous genotypes was always the shortest, except on chromosomes 1D, 3D, 4D, and 5D. When ordered by physical map, the full genome was 5757.77, 4712.47 and 4860.15 cM in length. As the marker orders were the same among the three maps, the difference on map length was caused by the impact of genotyping error on recombination frequency estimation. Replicate 2 formed a much shorter map than did replicate 1, which indicated that data quality of replicate 2 was better than that of replicate 1. The full genome ordered by nnTwoOpt was 4290.51, 4346.14 and 3817.46 cM in length for replicate 1, replicate 2 and non-erroneous genotypes of the JB population, much larger than counterparts of the YZ population (Table 3). Chromosome length from the non-erroneous genotypes was also the shortest in the JB population. The difference in map length between the non-erroneous genotypes and replicate 1 or replicate 2 became much larger, because of the higher genotyping error rate in the JB population. Upon being ordered by physical map, the full genome was 16001.36, 16192.33 and 15185.63 cM in length. The high error rate resulted in extremely long maps. Data quality of replicate 1 was better than that of replicate 2, resulting in a relatively shorter map. Although the non-erroneous method was applied, the map was still long, probably due to the marker order difference between the true linkage and physical maps.

**Table 2:** Comparison of map length in cM between genetic linkage maps ordered by nnTwoOpt and physical map in the Yangxiaomai×Zhongyou9507 RIL population

Chr.	Ordered by nnTwoOpt^a^			Ordered by physical map^b^		
	Replicate 1^c^	Replicate 2^d^	Non-err.^e^	Replicate 1	Replicate 2	Non-err.
1 A	154.11	153.00	151.83	161.32	162.69	161.00
1B	140.24	138.93	137.92	143.76	142.20	141.34
1D	252.90	251.30	252.14	669.40	634.80	667.58
2 A	191.57	193.10	189.44	215.68	210.70	214.32
2B	171.77	169.22	168.70	179.58	179.60	174.91
2D	167.35	167.89	166.78	167.47	169.60	166.88
3 A	230.02	229.01	227.44	250.44	254.11	250.86
3B	161.73	162.15	161.31	171.16	171.71	174.08
3D	226.31	231.28	226.71	259.66	246.63	256.87
4 A	226.79	226.39	225.66	276.37	276.76	273.44
4B	122.77	118.11	116.48	132.24	127.78	126.34
4D	117.03	116.36	116.91	127.08	124.76	127.37
5 A	290.21	289.12	286.74	336.73	329.72	328.07
5B	214.29	213.60	209.46	226.84	227.56	224.29
5D	256.54	258.39	258.12	256.55	260.44	258.13
6 A	96.11	96.10	94.64	96.57	98.37	97.06
6B	167.30	165.39	163.69	183.74	182.55	181.87
6D	123.33	124.87	120.62	126.07	125.72	125.18
7 A	192.46	190.63	189.31	199.00	197.54	195.57
7B	175.16	175.08	173.10	194.66	189.18	190.47
7D	262.49	260.38	255.17	1383.46	400.03	524.52
Total	3940.48	3930.33	3892.17	5757.77	4712.47	4860.15

^a^ The map constructed by nnTwoOpt, where nearest neighbor was used for tour construction, and two-opt was used for tour improvement

^b^ The map with the same marker order of the physical map

^c^ The map using the first replication of genotyping

^d^ The map using the second replication of genotyping

^e^ The map using the non-erroneous genotypic data, i.e., all inconsistent genotypes between the two replications of genotyping are replaced by missing values

**Table 3:** Comparison of map length in cM between genetic linkage maps ordered by nnTwoOpt and physical map in the Jingshuang16×Bainong64 RIL population

Chr.	Ordered by nnTwoOpt^a^			Ordered by physical map^b^			
	Replicate 1^c^	Replicate 2^d^	Non-err.^e^		Replicate 1	Replicate 2	Non-err.
1 A	77.83	85.78	69.63		82.41	97.81	71.55
1B	152.35	158.12	137.41		878.06	768.72	552.21
1D	163.83	169.86	147.28		973.42	1091.55	668.88
2 A	225.77	219.42	206.04		819.72	814.55	1449.17
2B	176.98	187.28	163.93		196.15	199.29	169.83
2D	192.86	184.26	165.69		926.63	1016.80	819.39
3 A	238.00	242.51	195.82		413.84	427.54	292.22
3B	287.76	283.28	247.31		869.14	814.77	833.41
3D	148.64	145.78	133.20		140.19	144.99	128.88
4 A	165.08	169.66	144.04		1137.07	1137.07	785.84
4B	119.86	119.07	100.66		121.91	123.00	100.65
4D	72.24	70.54	67.83		72.94	71.55	67.83
5 A	344.76	329.20	295.25		535.54	503.53	474.31
5B	210.48	218.84	193.06		615.78	638.17	683.89
5D	396.21	408.17	366.41		3696.93	2821.23	2832.06
6 A	159.37	156.94	142.30		300.85	303.35	290.07
6B	219.82	231.72	203.47		808.31	867.02	706.72
6D	119.74	125.82	110.90		119.74	131.66	110.90
7 A	252.56	261.29	229.28		1324.21	2229.52	2287.61
7B	224.46	231.36	198.35		812.40	889.56	848.31
7D	341.92	347.24	299.62		1156.12	1100.55	1011.90
Total	4290.51	4346.14	3817.46		16001.36	16192.23	15185.63

^a^ The map constructed by nnTwoOpt, where nearest neighbor was used for tour construction, and two-opt was used for tour improvement

^b^ The map with the same marker order of the physical map

^c^ The map using the first replication of genotyping

^d^ The map using the second replication of genotyping

^e^ The map using the non-erroneous genotypic data, i.e., all inconsistent genotypes between the two replications of genotyping are replaced by missing values

**Fig. 1:**
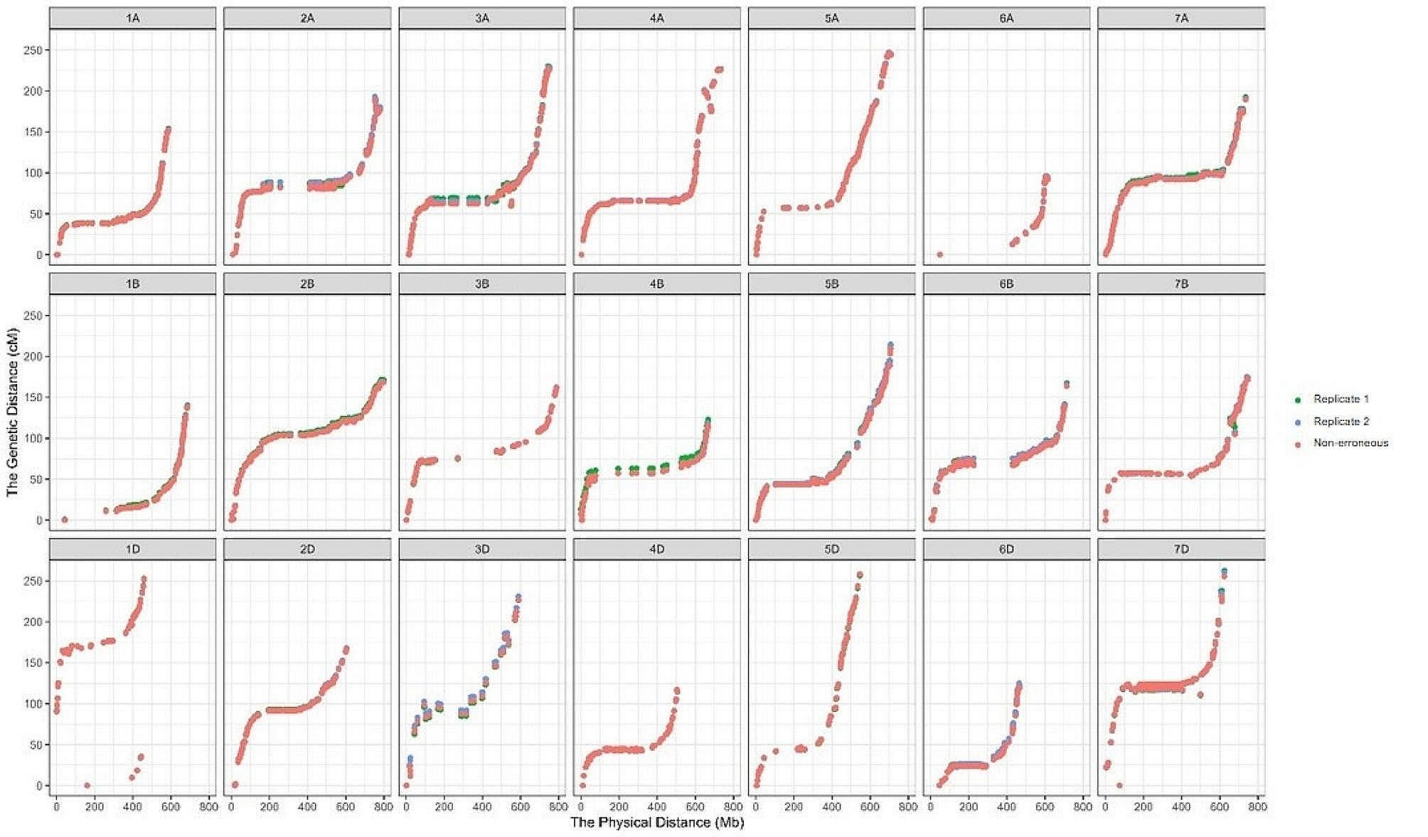
Collinearity of marker orders between linkage and physical maps in the Yangxiaomai×Zhongyou9507 RIL population. Different colors represent the source data for linkage map constructions, i.e., the first replication of genotyping (green dots), the second replication of genotyping (blue dots), and non-erroneous genotypes (red dots)

**Fig. 2:**
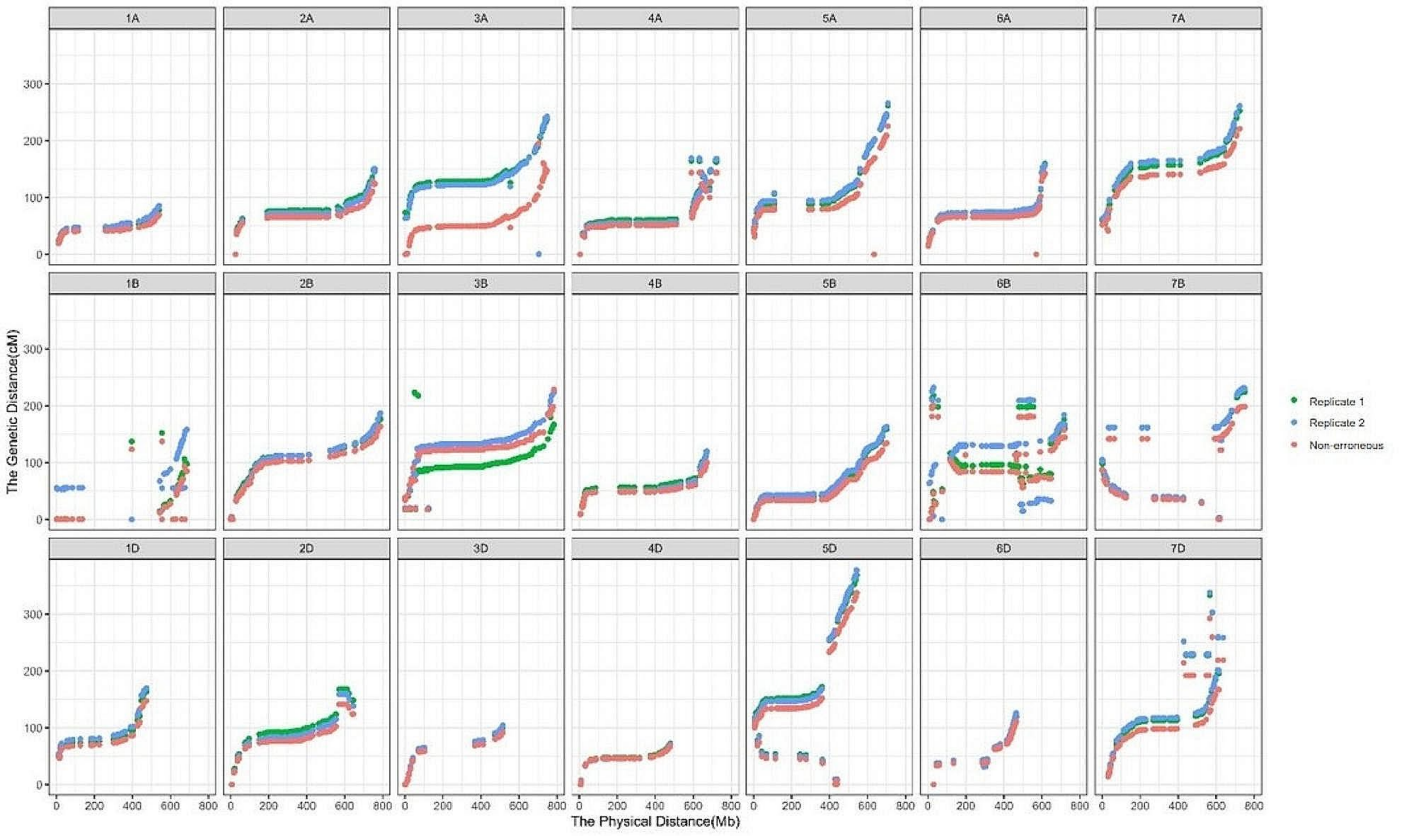
Collinearity of marker orders between linkage and physical maps in the Jingshuang16×Bainong64 RIL population. Different colors represent the source data for linkage map constructions, i.e., the first replication of genotyping (green dots), the second replication of genotyping (blue dots), and non-erroneous genotypes (red dots)

Collinearity of marker order between linkage and physical maps was shown in Fig. 1 for the YZ population and in Fig. 2 for the JB population by using R-package ggplot2 [34]. For population YZ, marker orders in the three linkage maps and physical map had high collinearity across the 21 chromosomes, and the difference among the three linkage maps was minor (Fig. 1). The downward trend of the non-erroneous map could still be observed on chromosomes 2 A, 4B, and 7 A, reflecting the shorter map from the non-erroneous genotypes. For population JB, lower collinearity of marker order between linkage and physical maps was observed, especially on chromosomes 1B, 5D, 6B, and 7 A (Fig. 2). Improvement of map length by the non-erroneous method was significant on all chromosomes except chromosome 3D.

Table S3 provided the Pearson correlation coefficient between linkage and physical maps constructed using different genotypic data in the two populations. For population YZ, the average correlation coefficient across all chromosomes was 94.69, 95.92 and 95.18% for replicate 1, replicate 2 and non-erroneous genotypes. Correlation coefficient was always higher than 90% except on chromosomes 1D, 6D, 7B and 7D. Correlation coefficients were much lower in population JB, and the average value across chromosomes was 75.05, 74.26 and 78.95% for replicate 1, replicate 2 and non-erroneous genotypes. The non-erroneous method improved the correlation coefficient, especially on chromosomes 3D, 4 A and 6B.

### Linkage maps constructed using different proportions of repeated genotyping individuals

Figure S1 shows the error rate of genotypic data in the A genome of population JB for different proportions of repeated genotyping individuals with a step size of 5%. As the non-erroneous method was applied for the repeated genotypes, rates of 01, 02, 12, and total errors decreased with the increasing of repeated proportion. At the same time, the missing rate increased, because detected errors were replaced by missing values. Similar trend was also observed in the B and D genomes.

Length of linkage maps using genotypic data with different proportions of repeated genotyping individuals was shown in Fig. S2, averaged from three replications of sampling. The rightmost column corresponded to the non-erroneous map. It could be seen intuitively that the corrected map (i.e., 5 to 50% repeated) was shorter than the original map (i.e., 0% repeated), but longer than the non-erroneous map (i.e., 100% repeated). Interestingly, when the repeated proportion was 30%, map length is the smallest among levels of 5 to 50%, which was closest to length of the non-erroneous map. Although error rate decreased with the increasing of repeated genotyping individuals, the map length expanded when more than 30% individuals were genotyped repeatedly. The reason may be the increasing missing rate with the increased repeated proportion. A high missing rate also decrease map quality, which is consistent with the results of the DH population experiment simulated by [12]. Therefore, if it is impossible to genotype all individuals repeatedly, 30% is recommended in repeated genotyping, which has a balance between error and missing rates.

### Comparisons of genetic maps constructed using genotypic data corrected by the EC and GC methods

The distribution of SNPs and linkage map information using the genotypes corrected by the EC and GC methods were given in Table 4 for the two populations. For population YZ, the full genome corrected by the EC method was 3347.90 and 3371.26 cM in length for replicate 1 (denoted by EC 1) and replicate 2 (denoted by EC 2), respectively, 592.58 and 559.07 cM shorter than the corresponding maps for original genotypic data. The full genome corrected by the GC method was 3189.35 and 2178.68 cM in length for replicate 1 (denoted by GC 1) and replicate 2 (denoted by GC 2), which was 1751.13 and 1751.65 cM shorter than the original map. For population JB, the full genome was 3819.02 and 3807.80 cM in length for EC 1 and EC 2, which was 471.49 and 538.34 cM shorter than the original map. The full genome was 2323.70 and 2311.91 cM in length for GC 1, GC 2, which was 1966.81 and 2034.23 cM shorter than the original maps. Length contraction by GC was much more significant than that by EC, but the map length corrected by EC was closer to the non-erroneous map length.

**Table 4:** Length of genetic linkage maps in cM using genotypic data corrected by the EC and GC methods in the two RIL populations

Chr.	Yangxiaomai×Zhongyou9507 RIL population				Jingshuang16×Bainong64 RIL population			
	EC 1^a^	EC 2^b^	GC 1^c^	GC 2^d^	EC 1	EC 2	GC 1	GC 2
1 A	127.47	130.38	98.46	98.03	73.20	78.97	69.91	71.15
1B	127.05	126.21	93.65	93.26	133.64	135.11	67.42	67.34
1D	211.92	212.34	119.16	121.79	148.41	147.34	77.63	76.11
2 A	158.34	157.83	97.65	98.11	203.96	203.42	145.40	155.28
2B	158.35	154.09	103.79	104.86	158.68	164.81	106.59	112.33
2D	149.85	148.74	104.46	103.48	185.70	175.34	107.10	109.67
3 A	205.42	207.57	109.66	110.45	220.10	218.03	108.53	105.20
3B	148.18	148.81	103.35	101.65	250.66	242.66	103.80	100.11
3D	190.50	201.46	103.15	106.17	127.19	132.37	85.11	81.88
4 A	189.89	190.76	131.11	126.54	154.23	155.23	126.99	119.38
4B	109.04	105.18	75.63	68.69	112.83	104.63	92.58	82.52
4D	93.73	93.91	90.73	91.15	67.88	64.39	65.67	64.47
5 A	208.37	209.10	130.37	130.73	291.23	277.26	162.81	160.63
5B	196.02	198.54	129.33	127.20	199.85	204.75	155.79	143.03
5D	212.43	214.95	129.41	129.46	326.53	314.06	133.48	136.35
6 A	81.48	82.11	52.67	52.00	126.12	125.04	84.02	80.63
6B	143.39	144.55	101.19	99.25	195.38	210.92	139.30	142.21
6D	101.65	103.24	68.33	68.07	111.80	114.01	90.47	92.15
7 A	163.47	163.80	119.65	118.22	225.27	231.10	133.80	133.10
7B	155.14	158.15	88.90	90.68	213.02	218.54	101.25	112.81
7D	216.21	219.54	138.70	138.89	293.34	289.82	166.05	165.56
Total	3347.90	3371.26	2189.35	2178.68	3819.02	3807.80	2323.70	2311.91

^a^ The map using the first replication of genotyping corrected by the EC method

^b^ The map using the second replication of genotyping corrected by the EC method

^c^ The map using the first replication of genotyping corrected by the GC method

^d^ The map using the second replication of genotyping corrected by the GC method

Pearson correlation coefficient between the corrected map and physical map was given in Table S4. For population YZ, the correlation coefficient greatly varied from 76.06 to 99.97% for EC 1, from 83.79 to 99.96% for EC 2, from 82.61 to 100% for GC 1, and from 91.13 to 99.99% for GC 2 on different chromosomes. The average correlation coefficient was 95.03, 96.01. 97.51 and 98.12% for EC 1, EC 2, GC 1, and GC 2, respectively. Both the EC and GC methods improved the correlation coefficient between linkage and physical maps, compared with the original genotypic data. Pearson correlation coefficients between different linkage maps and non-erroneous map were given in Table S5 for population YZ and in Table S6 for population JB. The linkage maps included the maps from replicate 1, replicate 2, EC 1, EC 2, GC 1 and GC 2. For population YZ, average correlation coefficient from EC was the highest, followed by GC and the original data sets (Table S5). For population JB, map from EC had similar or higher correlation coefficient than the map from the original data except on chromosome 3D. Map from the GC method had similar or lower correlation coefficient than did the original data except on chromosomes 1 A and 6B (Table S6). Generally speaking, in both populations, EC had higher correlation coefficient with the non-erroneous map than did GC and the original genotypes.

### Results in simulated populations

Length of linkage maps using original simulated data and genotypes corrected by the EC and GC methods in simulated chromosomes was given in Table 5. No matter whether genotypes were corrected or not, map length increased with the increasing of error rate. When simulated length was 100 cM, map using original genotypes ranged from 99.23 to 615.62 cM in length when error rate ranged from 0 to 5%; maps using genotypes corrected by the EC method ranged from 94.19 to 154.19 cM in length; maps using genotypes corrected the GC method ranged from 62.45 to 126.97 cM in length. When simulated length was 200 cM, map using original genotypes ranged from 199.81 to 1357.40 cM when error rate ranged from 0 to 5%; maps using genotypes corrected by the EC method ranged from 189.81 to 353.12 cM in length; maps using genotypes corrected the GC method ranged from 124.67 to 190.54 cM in length. It was concluded that if error correction was not conducted, map length was doubled when error rate was 1%, for both simulated chromosome length of 100 and 200 cM. Both error correction methods reduced the map length, and GC resulted in a shorter map than did EC. But map length from GC was significantly underestimated when error rate was smaller than 2% for map length of 100 cM and 5% for map length of 200 cM. For example, when error rate was 1%, map length from GC was only 73.89 and 136.70 cM, compared with predefined length of 100 and 200 cM. At this error rate, map length from EC was 89.20 and 197.84 cM, which was closer to the true values.

**Table 5:** Average length of genetic linkage maps using genotypic data corrected by the EC and GC methods at different genotyping error rates in the two simulated chromosomes

Map length (cM)	Error rate (%)	Original (cM)^a^	EC (cM)^b^	GC (cM)^c^
100	0	99.23	94.19	62.45
	0.5	148.61	95.23	69.83
	1	198.31	89.20	73.89
	2	299.95	103.74	87.83
	3	409.62	115.19	104.51
	5	615.62	154.19	126.97
200	0	199.81	189.81	124.67
	0.5	304.79	192.92	130.87
	1	412.64	197.84	136.70
	2	636.39	217.28	149.84
	3	877.13	249.58	162.22
	5	1357.40	353.12	190.54

^a^ The map using the original genotypic data

^b^ The map using genotypic data corrected by the EC method

^c^ The map using genotypic data corrected by the GC method

Table S7 provided the Pearson correlation coefficient of marker orders using different genotypic data with the predefined order. No matter genotypes were corrected or not, correlation coefficient decreased with the increasing of error rate. When simulated length was 100 cM, correlation coefficient using original genotypes ranged from 99.9657 to 99.1756% when error rate ranged from 0 to 5%; correlation coefficient using genotypes corrected by the EC method ranged from 99.9505 to 99.9316%; correlation coefficient using genotypes corrected by the GC method ranged from 99.9877 to 99.9874%. When simulated length was 200 cM, correlation coefficient using original genotypes ranged from 99.9975 to 96.6721% when error rate ranged from 0 to 5%; correlation coefficient using genotypes corrected by the EC method ranged from 99.9996 to 99.0349%; correlation coefficient using genotypes corrected by the GC method ranged from 99.9990 to 99.9980%. Both error correction methods improved correlation coefficient, and the difference between EC and GC was minor. Genotyping error had a more obvious impact on correlation coefficient for map length of 200 cM than did map length of 100 cM.

### Accuracy of error correction by EC and GC methods

**Fig. 3:**
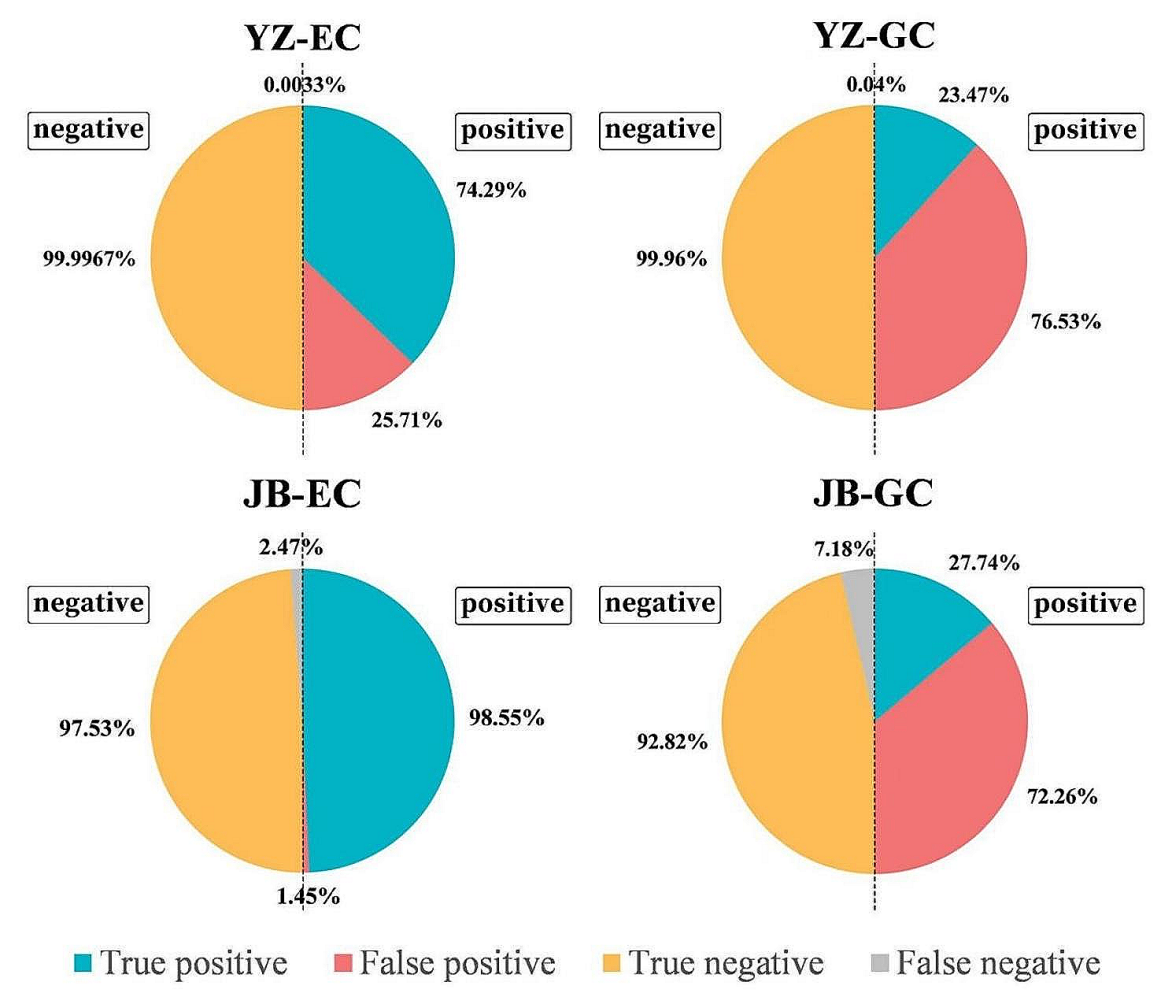
True positive, false positive, true negative and false negative rates of genotyping error correction by the EC and GC methods in two wheat RIL populations. YZ represents the Yangxiaomai×Zhongyou9507 RIL population, and JB represents the Jingshuang16×Bainong64 RIL population. Area of each circle is 2. The left half is the total percentage of true negative (yellow) and false negative (gray), with the area of 1. The right half is the total percentage of true positive (blue) and false positive (red), with the area of 1

**Fig. 4:**
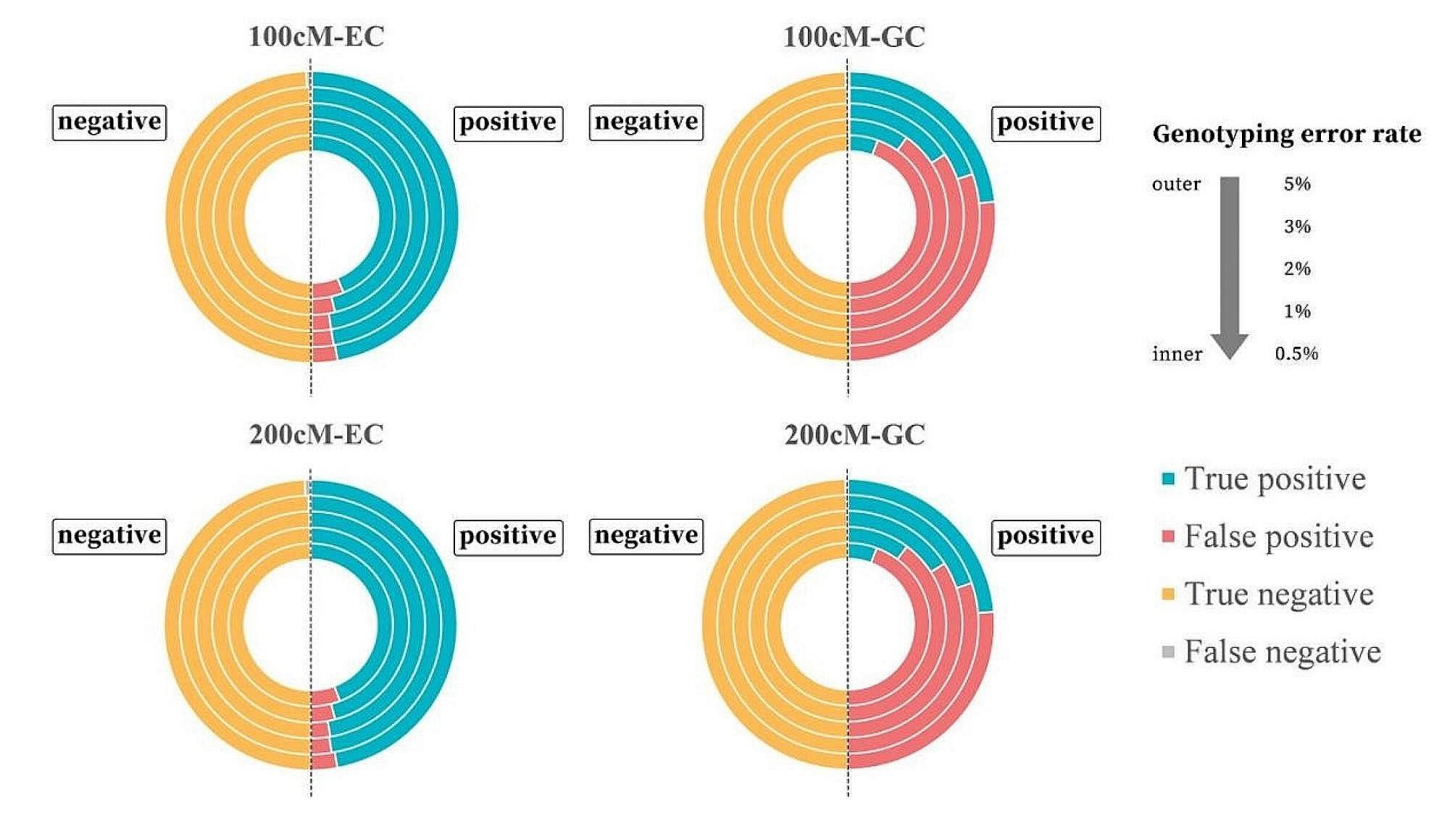
True positive, false positive, true negative and false negative rates of genotyping error correction by the EC and GC methods in the two simulated chromosomes at different genotyping error rates. Area of each circle is 2. The left half is the total percentage of true negative (yellow) and false negative (gray), with the area of 1. The right half is the total percentage of true positive (blue) and false positive (red), with the area of 1

Accuracy of EC and GC in the two actual RIL populations and simulated populations was calculated and shown in Figs. 3 and 4, representing by true positive, false positive, true negative and false negative rates. For a marker point, if there is a genotyping error, and the error correction method detects it, it is treated as true positive; if the method cannot detect it, it is treated as false negative. If there is no genotyping error, and the method regards it as a true genotype, it is treated as true negative; if the method regards it as an error, it is treated as false positive.

In population YZ, the true negative rate of the EC method was 99.9967%, while the true positive rate was 74.29%. The true negative of the GC method maintained well, reaching 99.96%, but the true positive rate was only 23.47%, which was far lower than that of the EC method. In population JB, the true positive rate of the EC method was as high as 98.55%, and the true negative rate was 97.53%. In contrast, the true negative rate of the GC method was 92.82%, but the true positive rate was only 27.74% (Fig. 3). In conclusion, for both RIL populations, the EC method had larger true negative and true positive rates than did the GC method. The false negative and false positive rates of EC were lower than that of GC.

For both simulated chromosome lengths and correction methods, true negative and true positive rates decreased with the increasing of error rate, while false negative and false positive rates increased. Difference on true negative and false negative rates between the EC and GC methods was minor, but EC had higher true positive and lower false positive rates than did GC at each error rate (Fig. 4). For example, when error rate was 5%, true negative and true positive rates of the EC method were 98.73 and 94.13%, while rates of the GC method were 99.44 and 47.25%. False negative and false positive rates of the EC method were 1.27 and 5.87%, while rates of the GC method were 0.56 and 52.75%. The high false positive rate of the GC method is an important reason of the underestimated map length. In other words, many accurate genotypes are treated as errors by the GC methods, resulting in a shorter map compared with the true map.

## Discussion

### Error rate in the two wheat RIL populations

The two populations were both sequenced by the 15 K wheat Affymetrix SNP array, but their data quality was much different, especially in error rate. Total error rate in the whole genome of populations YZ and JB was 0.35 and 8.47% (Tables S1, S2), respectively. One reason of the high error rate in the JB population (F_6_ RILs) may be that the population was not completely homozygous, leading to a relatively high heterozygosity of individuals. Heterozygosity of the two replications was 3.95 and 4.95% in the JB population, compared to corresponding values of 2.26 and 2.30% in the YZ population. Another notable finding is that the 01 and 12 errors have higher rates compared to the 02 error, especially in the YZ population. This observation aligns with previous researches where homozygous genotypes were mistakenly classified as heterozygous. It is crucial to address these errors as they can significantly affect the downstream analyses [35]. Owing to the higher error rate, map quality of population JB was much poorer than that of population YZ, both in map length, correlation coefficient, and collinearity of marker orders between linkage and physical maps (Tables 1 and 2, S3, Figs. 1 and 2).

### Repeated genotyping improves the map quality

The non-erroneous method based on repeated genotyping individuals improved the map quality in both populations, and the degree of improvement was much larger in population JB. Most studies typically perform only one round of genotyping. However, if budget allows, repeated genotyping would be preferable. Find out the loci with inconsistent genotypes and report them as genotyping errors, which will be replaced by missing values, or corrected by reliable error correction software. Pool et al. and Davey et al. also indicated that locus with high error rate can be accommodated as deletion data and reduced by appropriate statistical correction [36, 37]. If it is not allowed to conduct repeated sequencing for all individuals, 30% is a recommended proportion for repeated sequencing, which provides a balance between error and missing rates, and results in a relatively reasonable map length (Figs. S1, S2).

Some exception was observed on some chromosomes of population YZ, where the non-erroneous map was slightly longer than the map from one replication, such as chromosomes 1 A, 3D, 4D, and 5D (Table 2). An important reason may come from the algorithm of the non-erroneous method. Insistent genotypes between the two replications were replaced by missing. So after error correction, correctly assigned genotypes in one replication may become missing ones, which reduce the map quality to some extent. But this phenomenon disappeared when the error rate was higher, as the positive effect of error correction covered the negative effect of missing data. In population JB, all chromosomes in the non-erroneous map were shorter than those from each replication (Table 3). The negative effect from the non-erroneous method can be solved by replacing the error data point by right genotypes. But it is hard to derive the right genotypes from two replications of genotyping, and improvement should be conducted on the non-erroneous method using the linkage information.

### Comparison between the EC and GC methods for error correction

Besides the non-erroneous method, this study conducted comparison of efficiency and accuracy for error correction between the EC and GC methods using actual and simulated populations. Both methods shortened the map length and improved the correlation coefficient between linkage and physical maps in all populations, especially when the error rate was high (Tables 3 and 4, S4). Map from the EC method was closer to the non-erroneous map, and GC method resulted in a shorter map. But different from repeated genotyping, error correction software may produce wrong corrections. In the simulation experiment, map length form the GC method was shorter than the predefined length when error rate was low. It hints that the GC method may be too sensitive and conduct hypercorrection. This conclusion was proved by the calculation of true positive, false positive, true negative and false negative rates shown in Figs. 3 and 4. False positive rate of GC was much higher than that of EC.

Genotyping errors often reduce the power of linkage and association analysis, while current system to detect and correct genotyping errors is not satisfied [7]. Error correction improves statistical ability, but the correction process itself is prone to mistakes, and if not done well, new errors may occur. Further research and technical improvements are needed to solve the challenges. Firstly, many existing studies only used simulated data or a small number of real samples for verification of the error-correction methods. By applying these methods for large-scale data sets, performance of error correction software can be evaluated, and the room for improvement can be determined. Secondly, more precise and efficient error correction algorithms need to be developed. The current error correction software usually relies on a single site or small fragments, but it is still difficult for large-scale genome data processing. More comprehensive error correction strategies based on global genome information and machine learning are expected to be developed. In addition, we can also consider to optimize the sequencing platform and related equipment to improve the accuracy of genotyping at the technical level. For example, the adoption of more advanced and accurate gene sequencing techniques may significantly reduce the error rate and provide more reliable, accurate and reusable data for genetic analysis.

### Strategy for construction of high-quality linkage map

In this study, nnTwoOpt is adopted for marker ordering, which has been proved to be effective no matter the marker number is large or small [13]. Maps ordered by physical map were compared with those ordered by nnTwoOpt (Tables 1 and 2; Figs. 1 and 2). For some chromosomes, map length and marker order had small difference between the two methods, for example, on chromosomes 1 A, 1B, 2B in population YZ, and chromosomes 1 A, 2B, 3D in population JB, and so on. But the difference was much larger on some of the other chromosomes, such as chromosomes 1D and 7D in population YZ, and chromosomes 1B, 1D, 2 A in population JB, and so on. This phenomenon was observed in both populations, and the consistence of physical and linkage orders varies among chromosomes and populations. Translocation, inversion, genetic diversity among varieties, and many other reasons will all cause the difference between linkage order and physical order in the reference variety. Therefore, physical map only provides a reference for linkage map construction. It is not recommended to order markers same as the physical map. A speedy and accuracy ordering method is necessary for linkage map construction, especially when the marker number is large.

By repeated genotyping, it is found that the YZ population had lower genotyping error rate than the JB population. Error correction is more urgent and significant in the JB population. But in studies with only one replication of genotyping, it is hard to determine the error rate accurately. Under this circumstance, map length and Pearson correlation coefficient between linkage and physical maps can give us some suggestions. In both actual and simulated populations, map length increased with the increasing of error rate, meanwhile, the correlation coefficient decreased. Researchers should pay more attention to genotyping errors when linkage map is extremely long or Pearson correlation coefficient is low.

Repeated genotyping individuals improve map quality on both map length and consistence with physical map, no matter all individuals or only part of them are sequenced repeatedly. But of cause, more budget is needed. Software packages for genotyping error correction can also improve linkage map to some extent. But false positives and false negatives may be produced during the correction procedure, leading to overcorrection or under-correction on some chromosome segments. It is recommended to conduct genotyping error correction during the process of linkage map construction. The researchers can select repeated genotyping or correction packages depending on their budget and acceptance level of false positives and false negatives in error correction.

## Conclusion

Genotyping errors reduce the quality of genetic linkage maps, and in particular lead to inflated map lengths and reduced correlation coefficients with physical maps. The higher the error rate is, the worse the map quality is. By replacing the inconsistent genotypes with missing values, the map length was shortened and the correlation coefficient between linkage and physical maps was improved. Map quality can be improved significantly by error correction software. Map length form the EC method was closer to the non-erroneous map, and the accuracy of EC in actual and simulated populations was more stable, compared with the GC method. Although map from the GC method was shorter than that of the EC method, false positive rate of GC was rather high, leading to too short map compared to the true values.

### Electronic supplementary material

Below is the link to the electronic supplementary material.

Supplementary Material 1

Supplementary Material 2

## Data Availability

The input files for linkage map construction in the two RIL populations were submitted together with the article as supplementary data sets.

## Abbreviations

SNP: single nucleotide polymorphisms
QTL: Quantitative trait locus
LD: Linkage disequilibrium
RIL: recombinant inbred line
YZ: Yangxiaomai × Zhongyou 9507
JB: Jingshuang 16 × Bainong 64
K-Opt: k-Optimal
TSP: traveling-salesman problem
EC: error correction method in QTL IciMapping
GC: Genotype-Corrector
