# Supplementary material for: Effect of genotyping errors on linkage map construction based on repeated chip analysis of two recombinant inbred line populations in wheat (Triticum aestivum L.)
Source: BMC Plant Biol. 2024 Apr 22;24:306. doi: 10.1186/s12870-024-05005-8 (PMC11034145; doi:10.1186/s12870-024-05005-8)
Supplement: Supplementary file 2 — Supplementary Material 2 [file 12870_2024_5005_MOESM2_ESM.docx]

**Table S1** Genotyping error rate and missing rate in the genotypic data of the Yangxiaomai×Zhongyou9507 RIL population.

| Chr. | Maker No. | 01 error rate (%) | 02 error rate (%) | 12 error rate (%) | Total error rate (%) | Missing rate (%) |
| --- | --- | --- | --- | --- | --- | --- |
| 1A | 210 | 0.18 | 0.00 | 0.09 | 0.27 | 0.95 |
| 1B | 240 | 0.18 | 0.00 | 0.07 | 0.25 | 0.83 |
| 1D | 95 | 0.22 | 0.00 | 0.13 | 0.35 | 1.14 |
| 2A | 296 | 0.23 | 0.00 | 0.11 | 0.34 | 1.12 |
| 2B | 296 | 0.27 | 0.00 | 0.11 | 0.38 | 1.05 |
| 2D | 175 | 0.11 | 0.00 | 0.13 | 0.24 | 0.67 |
| 3A | 238 | 0.15 | 0.00 | 0.11 | 0.26 | 0.84 |
| 3B | 157 | 0.16 | 0.00 | 0.09 | 0.25 | 0.74 |
| 3D | 43 | 0.83 | 0.00 | 0.22 | 1.05 | 1.86 |
| 4A | 290 | 0.14 | 0.00 | 0.08 | 0.22 | 0.73 |
| 4B | 145 | 0.16 | 0.00 | 0.15 | 0.31 | 1.27 |
| 4D | 90 | 0.25 | 0.00 | 0.06 | 0.31 | 0.80 |
| 5A | 214 | 0.14 | 0.00 | 0.12 | 0.26 | 0.92 |
| 5B | 364 | 0.34 | 0.00 | 0.11 | 0.45 | 1.10 |
| 5D | 81 | 0.19 | 0.00 | 0.06 | 0.25 | 0.79 |
| 6A | 81 | 0.22 | 0.00 | 0.10 | 0.32 | 1.27 |
| 6B | 227 | 0.12 | 0.00 | 0.10 | 0.22 | 0.85 |
| 6D | 149 | 0.48 | 0.00 | 0.14 | 0.62 | 1.15 |
| 7A | 348 | 0.25 | 0.00 | 0.10 | 0.35 | 1.07 |
| 7B | 238 | 0.28 | 0.00 | 0.23 | 0.51 | 1.71 |
| 7D | 296 | 0.31 | 0.00 | 0.17 | 0.48 | 3.43 |
| Whole genome | 4373 | 0.23 | 0.00 | 0.12 | 0.35 | 1.18 |

**Table S2** Genotyping error rate and missing rate in the genotypic data of the Jingshuang16×Bainong64 RIL population.

| Chr. | Maker No. | 01 error rate (%) | 02 error rate (%) | 12 error rate (%) | Total error rate (%) | Missing rate (%) |
| --- | --- | --- | --- | --- | --- | --- |
| 1A | 157 | 3.57 | 1.00 | 3.52 | 8.09 | 1.42 |
| 1B | 72 | 3.13 | 1.98 | 2.85 | 7.96 | 1.78 |
| 1D | 99 | 2.28 | 0.94 | 2.09 | 5.31 | 1.08 |
| 2A | 458 | 3.07 | 3.02 | 3.13 | 9.22 | 1.40 |
| 2B | 180 | 2.96 | 1.93 | 2.90 | 7.79 | 1.21 |
| 2D | 250 | 3.45 | 1.90 | 3.48 | 8.83 | 1.73 |
| 3A | 210 | 3.60 | 3.53 | 3.51 | 10.64 | 1.50 |
| 3B | 470 | 3.31 | 1.92 | 3.25 | 8.48 | 1.35 |
| 3D | 117 | 1.78 | 1.30 | 2.38 | 5.46 | 1.13 |
| 4A | 219 | 2.53 | 1.92 | 2.61 | 7.06 | 1.03 |
| 4B | 176 | 2.75 | 3.01 | 3.07 | 8.83 | 1.56 |
| 4D | 58 | 4.11 | 1.26 | 3.68 | 9.05 | 1.22 |
| 5A | 334 | 3.78 | 2.85 | 3.57 | 10.20 | 1.58 |
| 5B | 396 | 2.73 | 2.63 | 2.48 | 7.84 | 1.47 |
| 5D | 164 | 3.27 | 2.48 | 3.48 | 9.23 | 1.22 |
| 6A | 318 | 2.93 | 2.15 | 3.04 | 8.12 | 1.52 |
| 6B | 231 | 2.46 | 2.33 | 2.24 | 7.03 | 1.41 |
| 6D | 60 | 3.23 | 1.61 | 3.23 | 8.07 | 1.89 |
| 7A | 219 | 3.32 | 3.14 | 3.45 | 9.91 | 1.66 |
| 7B | 166 | 3.12 | 1.84 | 3.06 | 8.02 | 1.09 |
| 7D | 143 | 3.26 | 1.85 | 3.36 | 8.47 | 1.45 |
| Whole genome | 4497 | 3.09 | 2.31 | 3.07 | 8.47 | 1.42 |

**Table S3** Pearson correlation coefficient between the linkage and physical maps constructed using different genotypic data in the two RIL populations.

| Chr. | Yangxiaomai×Zhongyou9507 RIL population | | | Jingshuang16×Bainong64 RIL population | | |
| --- | --- | --- | --- | --- | --- | --- |
|  | Replicate 1^a^ (%) | Replicate 2^b^ (%) | Non-err.^c^ (%) | Replicate 1 (%) | Replicate 2 (%) | Non-err. (%) |
| 1A | 99.49 | 99.39 | 99.17 | 99.94 | 99.89 | 99.96 |
| 1B | 99.81 | 99.93 | 99.92 | 43.72 | 58.80 | 51.83 |
| 1D | 83.78 | 83.80 | 83.78 | 70.57 | 70.17 | 69.89 |
| 2A | 93.61 | 97.12 | 99.63 | 73.92 | 50.87 | 63.24 |
| 2B | 99.90 | 99.95 | 99.93 | 99.88 | 99.98 | 99.82 |
| 2D | 99.76 | 97.35 | 97.00 | 47.73 | 47.91 | 49.13 |
| 3A | 90.55 | 91.82 | 92.13 | 94.44 | 94.41 | 98.54 |
| 3B | 99.89 | 99.86 | 99.90 | 83.67 | 83.37 | 89.29 |
| 3D | 96.64 | 96.62 | 96.62 | 40.48 | 46.97 | 68.86 |
| 4A | 92.52 | 96.26 | 94.86 | 79.87 | 81.96 | 91.70 |
| 4B | 99.81 | 99.70 | 99.86 | 99.98 | 99.49 | 99.14 |
| 4D | 95.96 | 96.76 | 97.00 | 88.04 | 92.04 | 93.94 |
| 5A | 99.81 | 99.68 | 99.70 | 98.49 | 98.50 | 98.45 |
| 5B | 98.96 | 99.54 | 98.76 | 90.62 | 93.10 | 89.28 |
| 5D | 99.96 | 99.96 | 99.97 | 51.13 | 51.24 | 52.96 |
| 6A | 99.95 | 99.95 | 99.95 | 86.04 | 89.06 | 88.03 |
| 6B | 92.42 | 93.82 | 92.44 | 13.00 | 16.87 | 42.69 |
| 6D | 76.05 | 91.04 | 78.17 | 99.99 | 71.15 | 96.68 |
| 7A | 94.90 | 94.99 | 94.62 | 65.34 | 64.96 | 65.68 |
| 7B | 85.59 | 87.30 | 85.92 | 67.96 | 68.01 | 67.98 |
| 7D | 89.03 | 89.45 | 89.55 | 81.32 | 80.76 | 80.82 |
| Mean | 94.69 | 95.92 | 95.18 | 75.05 | 74.26 | 78.95 |

^a^ The map using the first replication of genotyping.

^b^ The map using the second replication of genotyping.

^c^ The map using the non-erroneous genotypic data, i.e., all inconsistent genotypes between the two replications of genotyping are replaced by missing values.

**Table S4** Pearson correlation coefficients between the linkage maps constructed using genotypic data corrected by the EC and GC methods and physical map in the two RIL populations.

| Chr. | Yangxiaomai×Zhongyou9507 RIL population | | | | Jingshuang16×Bainong64 RIL population | | | |
| --- | --- | --- | --- | --- | --- | --- | --- | --- |
|  | EC 1^a^ (%) | EC 2^b^ (%) | GC 1^c^ (%) | GC 2^d^ (%) | EC 1 (%) | EC 2 (%) | GC 1 (%) | GC 2 (%) |
| 1A | 99.20 | 99.42 | 99.70 | 99.76 | 99.92 | 99.92 | 99.94 | 99.97 |
| 1B | 99.87 | 99.93 | 99.99 | 99.98 | 50.95 | 38.49 | 93.73 | 94.54 |
| 1D | 83.18 | 83.79 | 99.94 | 99.97 | 69.37 | 70.35 | 93.07 | 69.32 |
| 2A | 94.31 | 97.23 | 99.99 | 99.55 | 73.90 | 50.72 | 94.32 | 68.90 |
| 2B | 99.90 | 99.94 | 99.98 | 99.99 | 99.83 | 99.93 | 99.94 | 100 |
| 2D | 99.56 | 98.48 | 96.91 | 97.37 | 48.05 | 47.85 | 98.18 | 99.83 |
| 3A | 91.97 | 92.84 | 85.31 | 91.13 | 94.24 | 94.07 | 99.87 | 99.68 |
| 3B | 99.88 | 99.88 | 99.97 | 99.90 | 83.85 | 82.84 | 94.92 | 94.48 |
| 3D | 96.63 | 96.64 | 99.96 | 99.79 | 87.89 | 96.44 | 91.99 | 99.28 |
| 4A | 95.41 | 96.76 | 99.68 | 99.76 | 89.95 | 91.85 | 92.03 | 94.95 |
| 4B | 99.84 | 99.45 | 99.83 | 99.99 | 99.40 | 99.48 | 99.88 | 99.66 |
| 4D | 96.86 | 96.24 | 92.72 | 99.37 | 89.01 | 88.06 | 98.63 | 91.09 |
| 5A | 99.67 | 99.86 | 99.86 | 99.97 | 98.49 | 98.50 | 99.99 | 99.79 |
| 5B | 99.26 | 99.49 | 99.06 | 99.09 | 91.44 | 92.33 | 96.39 | 98.61 |
| 5D | 99.97 | 99.96 | 99.94 | 99.99 | 51.30 | 51.25 | 99.84 | 99.84 |
| 6A | 99.95 | 99.95 | 100 | 99.95 | 90.01 | 92.67 | 90.22 | 99.32 |
| 6B | 93.64 | 93.47 | 99.42 | 97.42 | 13.46 | 17.71 | 91.24 | 89.26 |
| 6D | 76.06 | 91.51 | 96.26 | 96.91 | 99.98 | 70.06 | 93.47 | 93.46 |
| 7A | 94.55 | 94.56 | 99.70 | 93.83 | 66.25 | 65.35 | 95.60 | 95.82 |
| 7B | 86.29 | 87.15 | 96.91 | 92.03 | 67.90 | 67.99 | 99.94 | 99.91 |
| 7D | 89.59 | 89.64 | 82.61 | 94.68 | 81.08 | 81.35 | 96.34 | 96.94 |
| Mean | 95.03 | 96.01 | 97.51 | 98.12 | 78.39 | 76.06 | 96.17 | 94.51 |

^a^ The map using the first replication of genotyping corrected by the EC method.

^b^ The map using the second replication of genotyping corrected by the EC method.

^c^ The map using the first replication of genotyping corrected by the GC method.

^d^ The map using the second replication of genotyping corrected by the GC method.

**Table S5** Pearson correlation coefficients between linkage maps using different genotypic data and non-erroneous map in the Yangxiaomai×Zhongyou9507 RIL population. The genotypic data includes the first replication, the first replication corrected by the EC and GC methods, the second replication data, and the second replication corrected by the EC and GC methods.

| Chr. | Replicate 1^a^ (%) | EC 1^b^ (%) | GC 1^c^ (%) | Replicate 2^d^ (%) | EC 2^e^ (%) | GC 2^f^ (%) |
| --- | --- | --- | --- | --- | --- | --- |
| 1A | 99.99 | 99.95 | 98.92 | 99.99 | 99.93 | 99.12 |
| 1B | 99.99 | 99.93 | 99.86 | 99.99 | 99.95 | 99.85 |
| 1D | 100 | 99.91 | 95.40 | 100 | 99.90 | 95.16 |
| 2A | 90.79 | 91.94 | 92.21 | 90.79 | 99.86 | 98.94 |
| 2B | 99.99 | 99.95 | 99.54 | 99.99 | 99.95 | 99.54 |
| 2D | 100 | 99.96 | 99.21 | 100 | 99.97 | 99.28 |
| 3A | 99.96 | 99.94 | 99.58 | 99.96 | 99.96 | 99.63 |
| 3B | 99.99 | 99.92 | 98.69 | 99.99 | 99.95 | 98.70 |
| 3D | 99.99 | 99.62 | 92.48 | 99.99 | 99.92 | 95.27 |
| 4A | 100 | 99.94 | 99.27 | 100 | 99.96 | 99.38 |
| 4B | 99.93 | 99.80 | 99.55 | 99.93 | 99.95 | 99.68 |
| 4D | 100 | 99.87 | 98.79 | 100 | 99.84 | 98.93 |
| 5A | 100 | 99.97 | 99.61 | 100 | 99.98 | 99.60 |
| 5B | 99.99 | 99.98 | 99.80 | 99.99 | 99.99 | 99.78 |
| 5D | 100 | 99.98 | 98.84 | 100 | 99.98 | 98.87 |
| 6A | 69.37 | 99.92 | 99.68 | 69.37 | 99.93 | 99.68 |
| 6B | 99.98 | 99.83 | 98.42 | 99.98 | 99.90 | 98.16 |
| 6D | 100 | 99.97 | 99.29 | 100 | 99.91 | 99.48 |
| 7A | 99.98 | 99.79 | 99.88 | 99.98 | 99.84 | 99.86 |
| 7B | 99.97 | 99.96 | 98.59 | 99.97 | 99.97 | 98.67 |
| 7D | 99.96 | 99.80 | 94.66 | 99.96 | 99.83 | 94.11 |
| Mean | 98.09 | 99.52 | 98.20 | 98.09 | 99.93 | 98.65 |

^a^ The map using the first replication of genotyping.

^b^ The map using the first replication of genotyping corrected by the EC method.

^c^ The map using the first replication of genotyping corrected by the GC method.

^d^ The map using the second replication of genotyping.

^e^ The map using the second replication of genotyping corrected by the EC method.

^f^ The map using the second replication of genotyping corrected by the GC method.

**Table S6** Pearson correlation coefficient between linkage maps using different genotypic data and non-erroneous map in the Jingshuang16×Bainong64 RIL population. The genotypic data includes the first replication, the first replication corrected by the EC and GC methods, the second replication, and the second replication corrected by the EC and GC methods.

| Chr. | Replicate 1^a^ (%) | EC 1^b^ (%) | GC 1^c^ (%) | Replicate 2^d^ (%) | EC 2^e^ (%) | GC 2^f^ (%) |
| --- | --- | --- | --- | --- | --- | --- |
| 1A | 92.28 | 99.95 | 99.88 | 94.35 | 99.84 | 99.45 |
| 1B | 99.98 | 99.93 | 97.59 | 96.62 | 99.95 | 97.19 |
| 1D | 99.99 | 99.96 | 81.37 | 99.98 | 99.98 | 81.71 |
| 2A | 99.86 | 99.99 | 86.47 | 99.87 | 99.99 | 85.78 |
| 2B | 99.96 | 99.96 | 98.31 | 99.88 | 99.96 | 98.91 |
| 2D | 99.94 | 99.99 | 94.93 | 99.97 | 99.99 | 93.86 |
| 3A | 99.98 | 99.97 | 98.68 | 97.78 | 97.84 | 99.07 |
| 3B | 93.03 | 99.53 | 93.50 | 93.10 | 99.89 | 95.22 |
| 3D | 99.96 | 79.01 | 75.88 | 99.98 | 79.12 | 76.96 |
| 4A | 99.97 | 99.98 | 99.24 | 99.98 | 99.99 | 99.16 |
| 4B | 99.84 | 99.96 | 99.54 | 99.70 | 99.96 | 99.61 |
| 4D | 99.90 | 99.95 | 99.32 | 99.71 | 99.96 | 99.68 |
| 5A | 99.98 | 99.91 | 98.16 | 99.90 | 99.93 | 98.86 |
| 5B | 99.86 | 99.99 | 95.52 | 99.91 | 99.99 | 96.30 |
| 5D | 99.84 | 99.92 | 93.32 | 99.41 | 99.71 | 94.57 |
| 6A | 99.96 | 98.50 | 95.46 | 99.85 | 98.95 | 95.36 |
| 6B | 80.83 | 95.29 | 90.82 | 79.07 | 99.98 | 79.29 |
| 6D | 99.91 | 99.92 | 99.32 | 99.75 | 99.75 | 99.04 |
| 7A | 99.91 | 99.94 | 92.20 | 99.94 | 99.95 | 90.93 |
| 7B | 99.98 | 99.99 | 96.63 | 99.98 | 99.99 | 97.03 |
| 7D | 99.95 | 99.93 | 95.24 | 99.96 | 99.85 | 95.50 |
| Mean | 98.33 | 98.65 | 94.35 | 98.03 | 98.79 | 93.98 |

^a^ The map using the first replication of genotyping.

^b^ The map using the first replication of genotyping corrected by the EC method.

^c^ The map using the first replication of genotyping corrected by the GC method.

^d^ The map using the second replication of genotyping.

^e^ The map using the second replication of genotyping corrected by the EC method.

^f^ The map using the second replication of genotyping corrected by the GC method.

**Table S7** Pearson correlation coefficient of marker orders using genotypic data corrected by the EC and GC methods at different genotyping error rates with the predefined marker order in the two simulated chromosomes.

| Map length (cM) | Error rate (%) | Original (%)^a^ | EC (%)^b^ | GC (%)^c^ |
| --- | --- | --- | --- | --- |
| 100 | 0.5 | 99.9657 | 99.9505 | 99.9877 |
|  | 1 | 99.9926 | 99.9975 | 99.9928 |
|  | 2 | 99.9962 | 99.9968 | 99.9880 |
|  | 3 | 99.8977 | 99.9927 | 99.9891 |
|  | 5 | 99.1756 | 99.9316 | 99.9874 |
| 200 | 0.5 | 99.9975 | 99.9996 | 99.9990 |
|  | 1 | 99.9956 | 99.9986 | 99.9981 |
|  | 2 | 99.4054 | 99.9973 | 99.9987 |
|  | 3 | 97.8324 | 99.9175 | 99.9977 |
|  | 5 | 96.6721 | 99.0349 | 99.9980 |

^a^ The map using the original genotypic data.

^b^ The map using genotypic data corrected by the EC method.

^c^ The map using genotypic data corrected by the GC method.


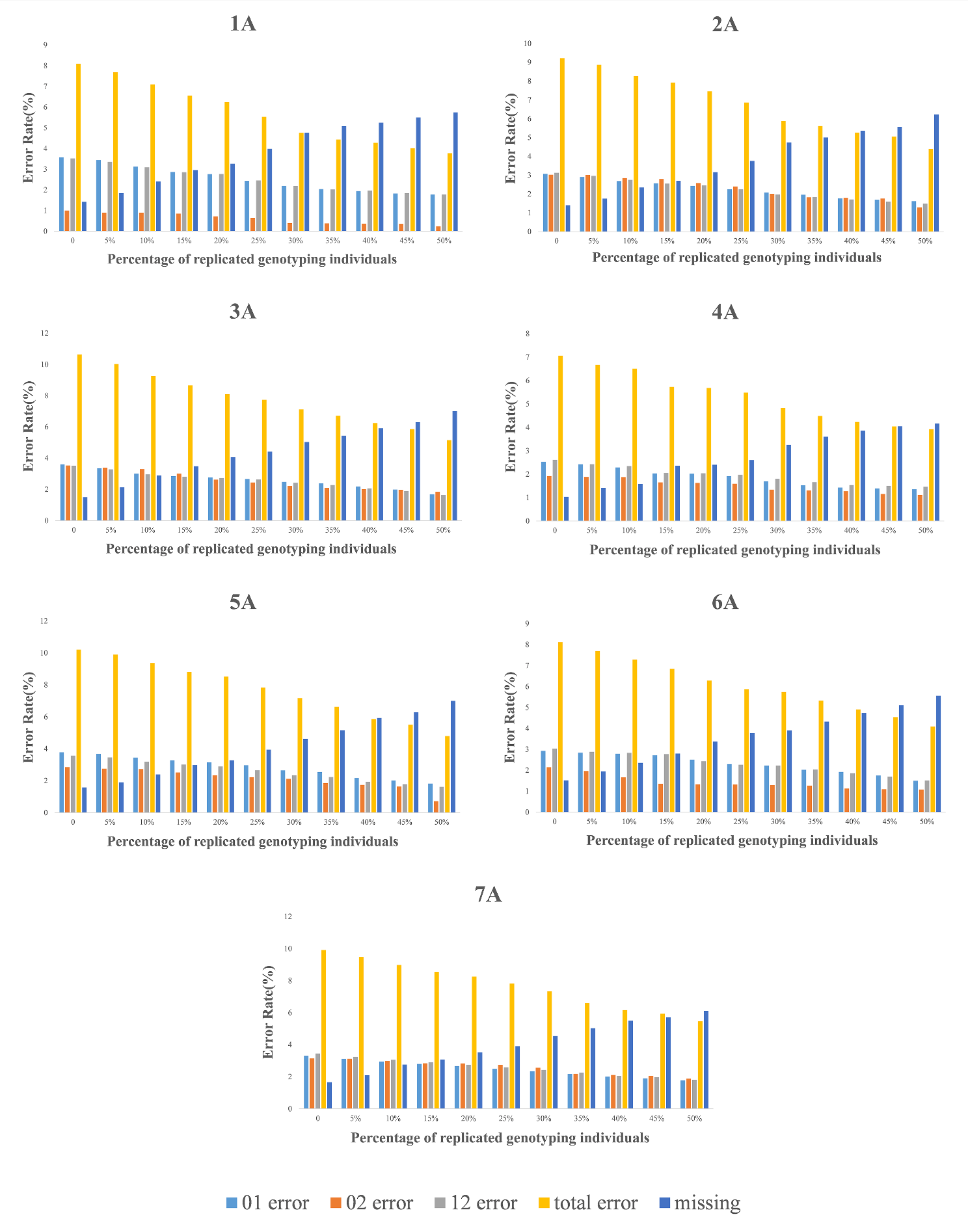


**Fig. S1** Error rate of genotypic data with different levels of repeated genotyping individuals in the A genome of the Jingshuang16×Bainong64 RIL population. Different bars represent different types of genotyping errors, i.e., 01 error (light blue), 02 error (orange), 12 error (gray), total error (yellow), and missing (dark blue). Total error is the sum of 01, 02 and 12 errors.

**Fig. S2** Length of genetic linkage maps constructed using genotypic data with different proportions of repeated genotyping individuals. The last bar (red) is the map length using non-erroneous data, i.e., all individuals were genotyped twice and different genotypes between the two replications are replaced by missing values.
